# Pilot-Scale Fermentation of *Pseudoalteromonas* sp. Strain FDHY-MZ2: An Effective Strategy for Increasing Algicidal Activity

**DOI:** 10.3390/biology12111447

**Published:** 2023-11-17

**Authors:** Yuying Zhong, Wenhuang Zheng, Xinguo Shi, Yisong Guo, Qianqian Wang, Pin Lv, Jianfeng Chen

**Affiliations:** 1 Technical Innovation Service Platform for High Value and High Quality Utilization of Marine Organism, Fuzhou University, Fuzhou 350108, China; 18960905802@163.com (Y.Z.); 18859807686@163.com (W.Z.); gys1028320304@fzu.edu.cn (Y.G.); 208527271@fzu.edu.cn (Q.W.); 208527263@fzu.edu.cn (P.L.); 2 Fujian Engineering and Technology Research Center for Comprehensive Utilization of Marine Products Waste, Fuzhou University, Fuzhou 350108, China; 3 Fuzhou Industrial Technology Innovation Center for High Value Utilization of Marine Products, Fuzhou University, Fuzhou 350108, China; 4 College of Advanced Manufacturing, Fuzhou University, Jinjiang 362200, China

**Keywords:** harmful algal blooms (HABs), shake flask fermentation optimization, pilot-scale fermentation optimization, algicidal bacteria, *Pseudoalteromonas* sp. FDHY-MZ2, *Karenia mikimotoi*

## Abstract

**Simple Summary:**

Recently, the occurrence of harmful algal blooms (HABs) in coastal areas has increased rapidly, negatively impacting fishing resources, public health, and marine ecosystems. Certain microorganisms play a significant role in the termination of HABs. To exploit these algicidal bacteria effectively, it is imperative to amplify their algicidal ratio and devise efficient large-scale cultivation methods; however, research in these areas is underdeveloped. We previously identified *Pseudoalteromonas* sp. strain FDHY-MZ2, which exhibited significant algicidal activity against *Karenia mikimotoi*, a species globally acknowledged for forming HAB. To improve the algicidal efficacy of FDHY-MZ2, cultures were progressed from shaking flask conditions to small-scale (5 L) and pilot-scale fermentation (50 L). The optimal fermentation medium and conditions at a pilot-scale level were established, resulting in a substantial improvement in algicidal properties. This enhancement might be due to a rise in algal H_2_O_2_ production accompanied by increased cell membrane disintegration, pronounced damage to cell chlorophyll and algal photosynthesis, and severe protein degradation. These findings provide new insights into the previously unknown potential of systematically applied microbial agents for the treatment of HABs.

**Abstract:**

The role of microorganisms in effectively terminating harmful algal blooms (HABs) is crucial for maintaining environmental stability. Recent studies have placed increased emphasis on bio-agents capable of inhibiting HABs. The bacterium *Pseudoalteromonas* sp. strain FDHY-MZ2 has exhibited impressive algicidal abilities against *Karenia mikimotoi*, a notorious global HAB-forming species. To augment this capability, cultures were progressively scaled from shake flask conditions to small-scale (5 L) and pilot-scale (50 L) fermentation. By employing a specifically tailored culture medium (2216E basal medium with 1.5% soluble starch and 0.5% peptone), under precise conditions (66 h, 20 °C, 450 rpm, 30 L/min ventilation, 3% seeding, and constant starch flow), a notable increase in algicidal bacterial biomass was observed; the bacterial dosage required to entirely wipe out *K. mikimotoi* within a day decreased from 1% to 0.025%. Compared to an unoptimized shake flask group, the optimized fermentation culture caused significant reductions in algal chlorophyll and protein levels (21.85% and 78.3%, respectively). Co-culturing induced increases in algal malondialdehyde and H_2_O_2_ by 5.98 and 5.38 times, respectively, leading to further disruption of algal photosynthesis. This study underscores the unexplored potential of systematically utilized microbial agents in mitigating HABs, providing a pathway for their wider application.

## 1. Introduction

*Karenia mikimotoi* is known to form harmful algal blooms (HABs) in coastal areas worldwide [1] and causes serious damage to marine ecosystems by producing hemolytic and cytotoxic compounds. Over the past two decades, it has been implicated in numerous fish deaths [2], leading to considerable economic implications [3]. Various methods have been employed for HAB control, including physical, chemical, and biological methods [4]. The application of algicidal bacteria presents a viable strategy for HAB control [5]. Several promising bio-agents have been reported to restrain HABs; for instance, naturally occurring bacteria associated with plants or macroalgae have been shown to impact a broad array of red tide organisms [6]. In addition, an algicidal bacterial strain, *Alteromonas* sp., was discovered in *Procentrum donghaiense* blooms in the East China Sea [7], pointing toward its potential use in direct mitigation strategies. Additionally, 365 strains of algae-lytic bacteria were identified in recent years, with *Pseudomonas* sp. comprising 6.3% of the total strains [8]. Notably, 86.7% of *Pseudoalteromonas* sp. algaelytic bacteria exterminate algae via the secretion of algaelytic compounds [9]. Optimal conditions and media for propagating *Pseudomonas* sp. are species-specific according to prior reports. For instance, the culture of *Pseudomonas denitrificans* under specific conditions—with an inoculum level of 10%, an initial pH of 7.0, and a rotation speed of 260 rpm—significantly influenced the production of vitamin B_12_ [9]. An optimized shake flask fermentation medium and conditions for *Pseudomonas aeruginosa* JP802 revealed optimal parameters of 28 °C, pH 7.5, an inoculum level of 10%, a rotation speed of 270 rpm, and a ventilation of 4 L·min^−1^ [10]. Despite these various reports on optimizing *Pseudomonas* spp. fermentation, there has been limited focus on culture optimization and prolongation of *Pseudoalteromonas* sp.

High cell density cultivation can considerably enhance microbial biomass and product formation by achieving cellular concentrations substantially higher than those obtained through conventional cultivation [11,12]. In the development of industrial-scale production of fermented products, there are typically three phases: small scale, pilot scale, and verification of the fermentation process [13]. The temperature, concentration, and material residence time distribution in small-scale equipment differ from those in large-scale equipment, leading to an “amplification effect” [14]. Regrettably, current literature offers sparse reports on the development of algicidal strain resources and the application of bacterial agents. This scarcity is most pronounced concerning the optimization of algicidal culture conditions and fermentation processes on a pilot scale. The major gap predominantly concentrates on shake flask optimization [15,16] and small fermenters [17], impacting the feasibility of industrial adaptation. It is necessary to explore the best fermentation optimization process through stratified research.

Factors such as rotation speed and ventilation rate significantly influence dissolved oxygen levels in the culture medium and are fundamentally essential for bacterial growth and the synthesis of algae-soluble substances. The optimization of rotation speed and ventilation rate to optimize dissolved oxygen is crucial. Furthermore, the inoculum amount appears to affect the high-density fermentation of microorganisms [18]. A suitable feeding process can augment bacterial density while concurrently mitigating the inhibitory impact of toxic substances present in the fermentation liquid. A study examining optimizing conditions to attain higher biomass and antimicrobial activity in the marine bacterium *Paenibacillus polymyxa* L1-9 in a 50 L fermenter [19] obtained optimal results with an inoculum level of 8%, initiating with a rotating speed and ventilation capacity of 250 r/min and 3 L/min, respectively, and supplementing the system with 6 g/L of glucose after 13 h of growth.

In addition to calculating the algicidal rate, the impact of algicidal bacteria can also be seen through a series of physiological and biochemical changes in algal cells. Contemporary studies addressing the characterization of algal cell damage induced with stress-related alterations have focused on three primary echelons: the cellular level [20], enzymatic level [21], and photosynthetic level [22]. At the cellular level, alterations manifest as changes in chlorophyll content and cell numbers; for instance, toxic chemicals can induce a decrease in the chlorophyll content of *K. mikimotoi* by detrimentally affecting the cell structure and impeding chlorophyll synthesis [23,24]. Concerning cellular damage, pigment ratios (chlorophyll c/chlorophyll a) notably increase post-exposure to substances like BDE-47, a constituent of polybrominated diphenyl ethers [25]. Enzymatic changes typically occur during stress responses to external toxins, which generate reactive oxygen free radicals [26]. Two key enzymes, superoxide dismutase (SOD) and catalase (CAT) constitute the antioxidant defense system [27]. The activities of SOD and CAT in the HAB species *Phaeocystis globosa* and *Heterocurvium akashiwo* were stimulated at lower concentrations of toxins but were inhibited at higher levels [28,29]. The malondialdehyde (MDA) level reflects the cell membrane lipid peroxidation degree [30] and is frequently utilized as an indicator for the extent of algal cell damage [31,32]. At the photosynthetic level, tracking chlorophyll fluorescence parameters indirectly indicates changes in photosynthesis [33]. Under the influence of modified clay, both the maximum photochemical rate and photosynthetic performance index of algal cells significantly decrease [34,35].

FDHY-MZ2 (*Pseudoalteromonas* sp.) is a bacteria strain with potent algicidal activity against *K. mikimotoi*. Our previous study identified this strain from an algal bloom event in Tongxin Bay, Lianjiang County, Fujian Province, China [15]. This study aimed to improve the algicidal efficiency of strain FDHY-MZ2 using a shake flask and fermenter, potentially enabling the application of the resultant product in controlling harmful algal blooms in the field. Conditions were optimized to augment the yield of bacterial metabolites to provide substantial quantities of end products. Single factor and orthogonal experiments were considered to discern optimal media and conditions for the shake flask culture. Additionally, fermentation optimization was performed in 5 L and 50 L fermenters, with the effects of scale-up scrutinized to assess alterations in cellular, enzymatic, and photochemical levels. The fermentation process was adjusted and scaled to boost the algicidal properties of the strain and increase the efficiency of large-scale growth. The resulting culture may be used to produce bacterial powder, which could be utilized to immediately kill toxic algae [22], and the biomass could be used to immobilize algicidal compounds using solidified materials, allowing for the continual release of algicidal chemicals over long periods [5].

## 2. Materials and Methods

### 2.1. Algal Cultures and Algicidal Bacteria

The *K. mikimotoi* culture was obtained from the Center for Collections of Marine Algae at Xiamen University, China (strain CCM-083). It was cultivated in an f/2-Si medium prepared with natural seawater [36] at 20 ± 1 °C under a 14:10 h light/dark cycle with a light intensity of 100 μmol photons m^−2^∙s^−1^. The seed algal culture was previously treated with multiple antibiotics [37].

*Pseudoalteromonas* sp. FDHY-MZ2 was isolated from the red tide area in Tongxin Bay, Lianjiang County, Fujian Province, China. It was preserved in a 2216E medium (containing 5 g of peptone, 1 g of yeast extract, and 0.01 g of FePO_4_ in 1 L of natural seawater, with a pH range of 7.0–7.8) enriched with 25% (*v*/*v*) glycerol and maintained at −80 °C. This medium was also used for bacterial growth, with the strain being cultured for 24 h at 25 °C and shaken at a rate of 150 rpm. A detailed description of its algicidal capabilities is available in past literature [22].

### 2.2. Analysis of Algicidal Rate

The algicidal rate was calculated using the following equation [38]:

Algicidal rate (%) = (NC − NT)/NC × 100

NT represents the density of algal cells treated with the bacterial culture, and NC represents the algal cell density in the negative control [7]. The algicidal experiment was conducted three times, each time utilizing an algal culture in its logarithmic growth phase. The volume of the bacterial culture was determined with the ratio of bacteria to algae; for example, the volume ratio of bacteria to algae of 1% (*v*/*v*) implies adding 1 mL of the bacterial culture to 100 mL of the algal culture. The specific bacterial–algal ratios are detailed in the methods for each particular experiment. The control group had the same volumes of the sterile 2216E medium as in treatments with the bacteria culture. Both the treatment and the control group were cultured in a light incubator at 20 °C and 80 μmol·m^−2^·s^−1^. After set intervals (detailed within each specific experiment), a 1 mL sample was drawn from the agitated co-culture solution and swiftly fixed using Lugol’s iodine solution. Algal cell enumeration was performed using a 0.1 mL plankton counting chamber.

### 2.3. Single Factor and Orthogonal Design to Optimize Shake Flask Culture of Strain FDHY-MZ2

A single-factor experimental design was used to determine the optimal culture conditions for bacterial strain FDHY-MZ2. The bacterial cultures were added to 100 mL of 2216E media. To optimize the culture duration, samples were extracted at 12 h intervals over 0 to 120 h. Temperature optimization experiments were conducted at 20, 25, 30, 35, and 40 °C. The influence of pH on growth and algicidal activity was assessed using initial pH values of 5.0, 6.0, 7.0, 8.0, and 9.0. The effect of rotation speed was tested at 50, 100, 150, 200, and 250 rpm. Seeding volume was set to 1, 3, 5, 7, and 10%. The growth status (biomass) and algicidal effect (algicidal rate) were used to evaluate the efficacy of the optimized conditions. Sample groups were assayed in triplicate every 24 h with absorbance measured at 600 nm, indicative of the optical density of the bacterial cultures. For algicidal rate assessment, bacterial samples were added to algal cultures at a volume ratio of 1% (bacteria/algae).

To optimize medium composition for the FDHY-MZ2 strain, both growth rates and inherent algicidal activities were evaluated with varied nitrogen and carbon source additions within a basal medium (2216E excluding nitrogen and carbon sources). Possible nitrogen sources, each supplemented at 0.5% (*v*/*v*) within the 100 mL 2216E basal medium, included peptone, yeast extract, soybean peptone, corn powder, urea, calcium ammonium nitrate (CAN), ammonium chloride (NH_4_Cl), and sodium nitrate (NaNO_3_). Potential carbon sources for optimization were also introduced at 0.5% (*v*/*v*), including glucose, fructose, xylose, lactose, mannitol, soluble starch, bran, and sodium citrate. Cultures were cultivated within a temperature-constant oscillator set to 25 °C at 150 rpm for 24 h. The methodologies used for biomass and algicidal rate determination paralleled those previously described.

Based on the results of the single-factor experiment, the factors with the highest effects on the algicidal activity were selected to construct an experiment utilizing L_9_(4^3^) orthogonal arrays [39]. Four factors at three levels were considered (Table S1). Both carbon and nitrogen source levels were maintained at 1.0 ± 0.5%, while pH values were held at 8.0 ± 0.5. Fermentation periods encompassed 24, 36, and 48 h. These were deemed the most important factors based on the single-factor experiment, while conditions such as optimal fermentation temperature, rotation speed, and seeding volume were kept constant. Based on an L_9_(4^3^) orthogonal array design, nine experiments were performed in triplicate. The optimal combination was established through the identification of the highest values for OD_600_, dry weight, and algicidal rate.

### 2.4. Optimization of Small-Scale Fermentation (5 L Fermenter) Conditions

The optimized shake flask culture conditions and medium were replicated using a 5 L fermenter (BIOTECH-5BGZ, BAOXING BIO, Shanghai, China). The bacterial culture was inoculated into the optimized 2216E medium at 5% (*v*/*v*) and fermented at 25 °C with a ventilation rate of 2 L/min. To examine how varying the rotational speed affected bacterial growth and total carbohydrate consumption, rotational speeds of 150, 200, and 300 rpm were used. pH variations were investigated using initial pH values of 6.5, 7.5, and 8.5. Three feeding strategies were implemented to trace changes in total carbohydrate quantities over time: single carbon source delivery using soluble starch, single nitrogen source usage using peptone, and basal medium containing both carbon and nitrogen sources. Sampling was performed at 6-h intervals with instantaneous measurement of relevant parameters; these parameters (OD_600_, dry weight, pH, dissolved oxygen (DO), and algicidal rate), set at a bacteria-to-algae volume ratio of 0.05%, were employed to ascertain optimal conditions.

### 2.5. Optimization of Pilot Fermentation (50 L Fermenter) Conditions

Further optimization was performed with a 50 L fermenter (BL-BJ-50 L, BRAUN BIOENGINEERING EQUIPMENT, Zhenjiang, China), drawing on the optimized conditions defined in the 5 L fermenter. Algicidal rate (set at a bacteria-to-algae volume ratio of 0.0075%) and the OD_600_ value were measured. The optimization indicators included rotation speed, aeration volume, inoculation volume, and nutrient replenishment strategy. The rotational speed was tested at 150, 300, and 450 rpm. Aeration was set at 10, 30, and 50 L/min. Inoculation volumes were 1%, 3%, and 5%. The nutrient supplementation strategy involved providing either a three-fold concentrated complete medium or a carbon source solution employing soluble starch with an equivalent content. The temporal pacing for sampling and subsequent measurement parameters mirrored those outlined in Section 2.4. Experiments were replicated three times for the sake of accuracy and reliability.

### 2.6. Estimation of Protein, Oxidative, and Antioxidant Systems in Algal Cells

Protein extraction was performed using the Coomassie Brilliant Blue method [40]. The control group was grown in the f/2 medium, and differing algicidal treatments were applied in three separate groups. The first was subjected to a 0.3% (*v*/*v*) FDHY-MZ2 culture, developed from a shaking flask utilizing 2216E media. A second group, optimized in a shaking flask, was exposed to the 0.05% (*v*/*v*) shake-flask-optimized fermentation liquid. The third group, cultivated in a 50 L fermenter, was exposed to the 0.0075% (*v*/*v*) fermentation liquid of the optimized 50 L fermenter culture. The algicidal rate and other parameters were measured at 0, 3, 9, and 12 h after inoculation of the algicidal bacteria. Algal cells were harvested using centrifugation at 4 °C and 8000 rpm for 20 min. The pellets were then resuspended in 0.2 mL of an enzyme extraction buffer—a mixed solution of 0.0146 g of EDTA-2Na and 0.5 g of polyvinylpyrrolidone in 50 mL of H_2_O—and supplemented with 0.8 mL of a 0.15 mol/L NaCl solution and 5 mL of Coomassie Brilliant Blue (Beijing Solarbio Science & Technology, China). Subsequently, each component was homogenized through a biological sample homogenizer (HBR-6&24, Hengao Technology, Tianjin, China) for 5 m/s, with a 20 s vibration and 40 s rest, repeating this cycle 5 times after 10 min of freezing. The protein concentration was determined using a spectrometer (SP-756P, Spectrum Instruments, Shanghai, China), setting an absorbance value of 595 nm [41]. Absorbance values of protein standard solutions at concentrations of 20, 40, 60, 80, and 100 μg/mL were measured at 595 nm to construct a standard curve. The corresponding protein content was then inferred based on the absorbance.

For oxidative and antioxidant enzyme activity assessment, cells were collected using centrifugation as described above. The cell pellets were resuspended in 5 mL of Milli-Q ultrapure water and homogenized (HBR-6&24, Hengao Technology, Tianjin, China) according to the manufacturer’s instructions. Cell debris was subsequently discarded through centrifugation, carried out at 5000× *g* for 10 min at 4 °C. The supernatant was used for the measurement of antioxidant enzymes, including MDA, H_2_O_2_, SOD, and CAT. The above enzyme activities were measured with corresponding kits following the manufacturer’s instructions (Jiancheng Bioengineering Institute, Nanjing, China).

### 2.7. Determination of Chlorophyll a and Photosynthetic Parameters in Algal Cells

Both cellular chlorophyll content and the photosynthetic metrics of algal cells were investigated under exposure to bacteria cultured using varied strategies. The sampling methodology paralleled that outlined in Section 2.6. The method for measuring chlorophyll a content was adapted from ethanol extraction of chlorophyll [42]. Chlorophyll fluorescence was measured with a pulse-modulated chlorophyll fluorescence instrument, Water-PAM (Walz, Germany) [43], controlled with Wincontrol software. The maximum photochemical efficiency (Fv/Fm), effective photochemical efficiency (YII), and photoresponse curve (P-I curve) with the relative electron transport rate (rETR; micromole electrons per square meter per second) were measured using a pulse-modulated fluorimeter (PAM-2100, Waltz, Effeltrich, Germany). For the rETR measurement, eight consecutive light levels of 50, 100, 150, 300, 500, 1000, 1500, and 2000 μmol photons·m^−2^·s^−1^ were set, with each stage pausing for 10 s and being separated with a 0.8 s saturating pulse of 5000 µmol photons·m^−2^·s^−1^.

### 2.8. Statistical Analysis

The obtained data were analyzed using Excel, Origin 2019b, and SPSS Statistics v20 (ANOVA statical analyses). SPSS Statistics was also utilized for a Waller–Duncan (as delineated in the experimental analysis, Section 3.4) and LSD significance analysis (referenced in experimental analyses, Section 3.1 and Section 3.5). In the Waller–Duncan significance analysis, different lowercase letters indicate statistically significant differences between treatment groups under the same conditions, and the significance level is *p* < 0.05. In an LSD significance analysis by using SPSS Statistics, * represents *p* < 0.05, indicating a significant difference; ** represents *p* < 0.01, denoting an extremely significant difference; *** represents *p* < 0.001, suggesting an exceedingly significant statistical difference.

## 3. Results

### 3.1. Optimization of Shake Flask Culture and Medium for Strain FDHY-MZ2

**Figure 1:**
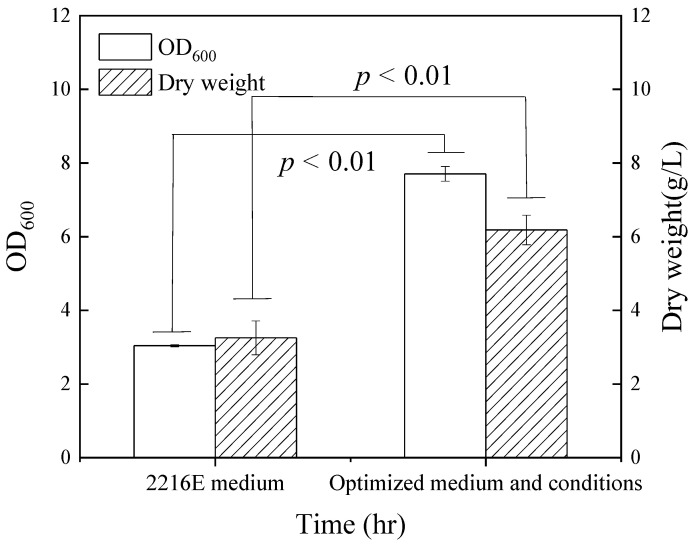
The OD_600_ value and dry weight of strain FDHY-MZ2 with 2216E medium and optimized cultivate (medium and conditions) in shake flask. Data represent the mean *± SD (standard deviation)* of triplicate.

To intensify the algicidal efficacy of the FDHY-MZ2 strain against *K. mikimotoi*, optimization of the shake flask culture was carried out. Taking into account the derived results of the single-factor experiments (Figure S1), optimal conditions were an initial pH of 7.5, a 24 h fermentation period, temperature of 20 °C, rotation speed of 150 rpm, and a 5% (*v*/*v*) inoculum as determined with biomass and algicidal rates. The preferred culture medium comprised soluble starch as the carbon provider and peptone as the nitrogen provider (Figure S2). Given that the primary function of algicidal bacteria is to directly eliminate algae, conditions rendering the highest algicidal rate were favored over those merely yielding the greatest biomass. Subsequently, an orthogonal experimental optimization encompassing four factors and three levels was initiated, based on single-factor optimization. The experiment factors, levels, and results are displayed in Table 1. An intuitive analysis, coupled with range testing, revealed combination No. 8 demonstrating the pinnacle of algicidal rate and bacterial density, with a remarkable 91.4% and an OD_600_ = 7.71, respectively (Figure S3): 1.5% inoculum, nitrogen source at 1.0%, pH = 7.5, and fermentation time of 48 h. The corresponding R-values deduced from the multi-factorial impact were fermentation time > nitrogen source concentration > carbon source concentration > pH value. The most potent combination comprised 0.5% peptone, 1.5% soluble starch, a pH value of 7.5, and a 48 h fermentation period, all established according to the optimal level (the level with the highest k value in this factor). In these optimized conditions, notable improvements were observed in dry weight and biomass. Both indices reported significant increases, with dry weight and biomass exhibiting a 1.9- and 2.54-fold rise, respectively, compared to the pre-optimization values (*p* < 0.01) (Figure 1).

### 3.2. Optimization of Fermenter Conditions for Scale-Up

#### 3.2.1. Small (5 L) Fermenter Condition Optimization

Based on existing literature, rotational speed, pH, and feeding strategy were chosen to optimize a 5 L fermenter set-up. At 300 rpm, the algicidal rate was higher than that at 150 rpm and 200 rpm, reaching 100% in 24 h (Figure S4). As the fermentation duration lengthened, the algicidal rate at 300 rpm remained stable, while the algicidal rate under the other two rotational speed conditions reached about 20%, maintaining a constant level. Biomass yield exhibited hardly any variation across all times regardless of the rotational speed. In the pH optimization, the culture group with a pH of 7.5 surpassed those at a pH of 6.5 and 8.5, denoted with superior biomass accumulation and algicidal rate (Figure S5).

**Figure 2:**
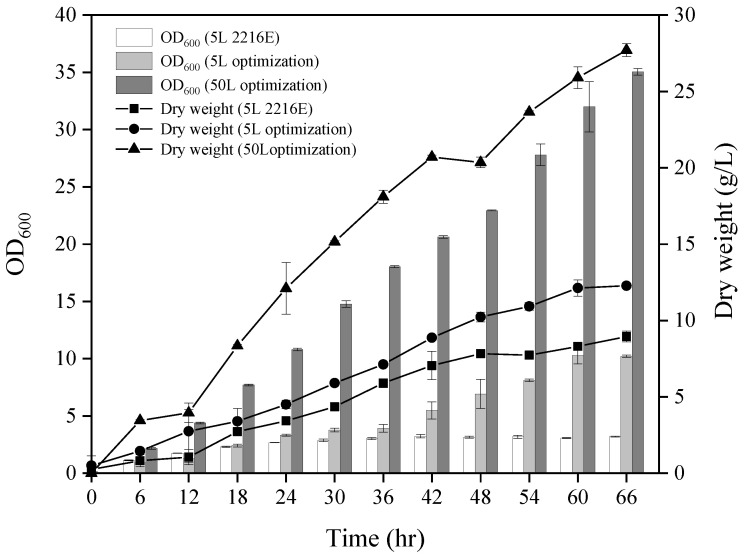
The OD_600_ value and dry weight of strain FDHY-MZ2 culture with 2216E 5 L, optimized 5 L, and 50 L optimization. Data represent the mean *± SD (standard deviation)* of triplicate.

In terms of the feeding strategy, biomass decreased by 6% every 6 h post 60 h of the culture with only nitrogen addition. The addition of both carbon and nitrogen resulted in an initial exponential rise in growth rate and a sustained higher OD_600_ value of 15.7. Although the biomass of the carbon source experimental group was higher after 78 h of fermentation, the complete culture medium experimental group reduced the consumption caused by operation while ensuring high biomass for a prolonged period. Accordingly, the most effective feeding strategy was found to incorporate the complete culture medium (Figure S6). The optimal conditions for small-scale fermentation (5 L) were a rotational speed of 300 rpm, unregulated pH, and a feeding strategy that included a comprehensive culture medium. Adopting these parameters yielded a 3.99 increase in OD_600_ compared to unoptimized fermentation (Figure 2); however, it did cause a delay in achieving a 100% algicidal rate due to a diminished bacteria-to-algae volume ratio. When this ratio was reduced to 0.025% (*v*/*v*), the fermentation broth achieved a 100% algicidal rate after 84 h. This modification led to a remarkable reduction in the lethal bacteria volume needed to achieve a 100% algicidal rate (decreasing from 1% to just 0.025%) (Figure S7).

#### 3.2.2. Large (50 L) Fermenter Condition Optimization

Applying the same approach as before, the conditions that enabled a fast 100% algicidal rate while maintaining stable growth and superior biomass yield were selected. Although the biomass at 300 rpm was higher than at 450 rpm before 48 h, it dropped rapidly in the later period while the 450 rpm speed displayed exponential growth within the first 60 h, sustaining a stable, high biomass state afterward. This speed consistently presented a higher algae dissolution rate than 300 rpm (100% faster) (Figure S8a,b). An evident increase and stabilization of dissolved oxygen in the fermentation tank were observed (>70%) upon increasing the rotation speed (Figure S9). Because of this, 450 rpm was selected as the optimal rotation speed for the 50 L fermenter.

Examining aeration, the biomass accumulation speed of the 30 L/min aeration group surpassed those of the 10 L/min and 50 L/min aeration groups. The algicidal rate of this group and the 50 L/min aeration group both reached 100% at 42 h, which is 6 h ahead of the 10 L/min aeration group (Figure S8c,d). For inoculum volume optimization, the biomass accumulation speed of the 3% inoculum group was higher than that at 1% and 5%. The algicidal rate of this group and the 5% inoculum group reached 100% after 42 h of fermentation, 6 h earlier than the 1% inoculum group (Figure S8e,f). Considering economic costs and equipment energy expenditure, when similar algicidal rates were yielded, 3% emerged as a more efficient inoculum volume compared to 5%. In the nutrient supplement optimization trial, the group supplemented with carbon sources exhibited significantly higher final biomass, roughly 1.6 times more than the groups without supplementation and those supplemented with a complete medium. This group reached a 100% algicidal rate at 36 h, which was 6 h and 12 h earlier than the non-supplemented group and the group supplemented with the complete medium, respectively (Figure S8g,h). Overall, the optimal parameters for the 50 L fermenter were set as a rotation speed of 300 rpm, aeration volume of 30 L/min, inoculation volume of 3%, and culturing with carbon source supplementation.

Under the optimal conditions obtained above (Table S2), FDHY-MZ2 was cultured in a 50 L fermenter and compared with the results of the 2216E basic medium culture in a 5 L fermenter, as well as the results of the optimized culture in a 5 L fermenter. Throughout the entire cultivation process, both the OD_600_ value and dry weight of the fermentation broth in the 50 L fermenter were significantly greater than those in the 5 L fermenter. After 66 h of the culture, the OD value of the fermentation broth in the 50 L fermenter was 3.43 times and 2.24 times that of the basic and optimized cultures in the 5 L fermenter, respectively. The dry weight of the fermentation broth in the 50 L tank was 3.09 times and 2.26 times that of the basic and optimized cultures in the 5 L fermenter, respectively (Figure 2). Algae dissolution was carried out with a bacteria-to-algae volume ratio of 0.025% (*v*/*v*), and a 100% algicidal rate was reached after 36 h of cultivation in the 50 L fermenter (Table S2).

### 3.3. Cellular Photosynthetic Pigment and Protein in K. mikimotoi to Optimized Pseudoalteromonas sp. FDHY-MZ2 Culture

**Figure 3:**
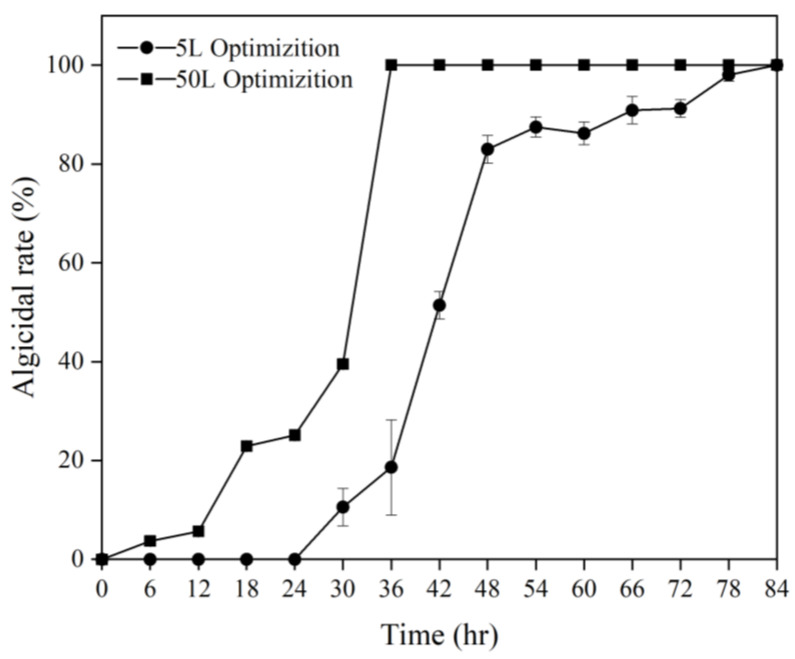
The algicidal rate of strain FDHY-MZ2 culture on *K. mikimotoi* with 5 L and 50 L optimization (bacteria–algae ratio, both 0.025%). Data represent the mean *± SD (standard deviation)* of triplicate.

**Figure 4:**
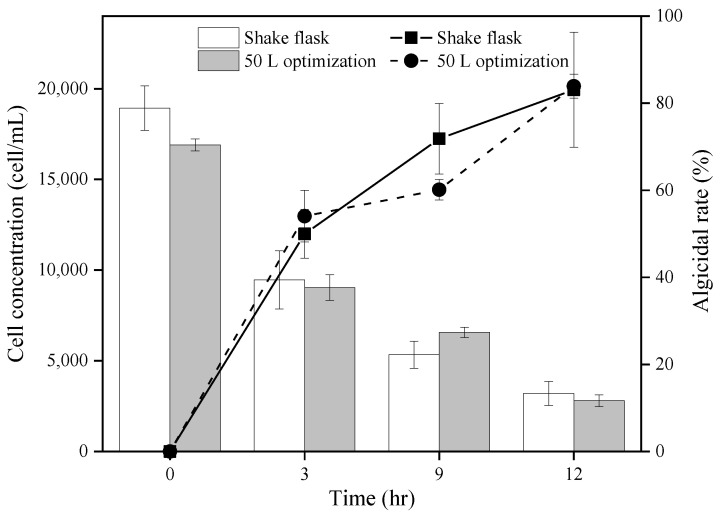
Results of cell concentration (column bar) and algicidal rate (black line) of co-culture with *K. mikimotoi* and different FDHY-MZ2 fermentation broth (shake flask and 50 L optimization; the bacterial-to-algal ratios were 0.3% and 0.075% (*v*/*v*), respectively). Data represent the mean *± SD (standard deviation)* of triplicate.

After optimizing the fermentation conditions, the bacterial-to-algal ratio capable of achieving a 100% algal dissolution rate within 24 h was determined; a 0.025% ratio was effective for a 50 L fermenter (Figure 3). When assessing photosynthetic parameters and enzymes, it is crucial that experiments are controlled to coincide with the photoperiod. For subsequent trials, bacterial-to-algal ratios ensuring a >80% algal dissolution rate within 12 h were selected. Gradient experiments revealed that treating 50 L of the optimized fermentation broth with a bacterial-to-algal ratio of 0.075% (*v*/*v*) caused the dissolution rate to surpass 80% within 12 h (Figure 4).

**Figure 5:**
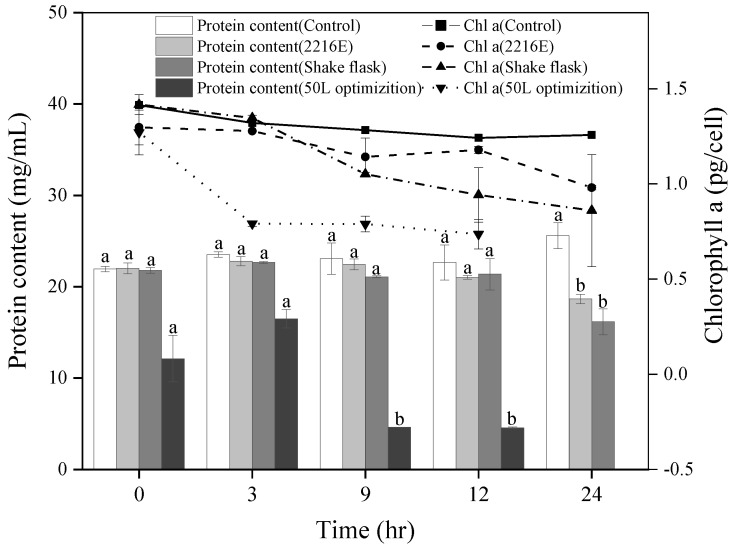
Protein and Chl a content of *K. mikimotoi* cell that co-ultured with different FDHY-MZ2 fermentation broth (2216E, shake flask, and 50 L optimization). Data represent the mean *± SD (standard deviation)* of triplicate. Different lowercase letters indicate statistically significant differences; the significance level is *p* < 0.05.

To investigate the influence of original and optimized FDHY-MZ2 algicidal cultures on *K. mikimotoi* photosynthetic pigments and protein, chlorophyll a and cellular protein were measured in three algal culture groups: those with 50 L fermenter optimized bacteria, those with unoptimized shaking-flask bacteria, and those without addition (control group) (Figure 5). For chlorophyll a, the quantity of cellular pigment decreased in both the group with optimized bacteria from the 50 L fermenter and the group with unoptimized shaking-flask bacteria. After 12 h of treatment, the cellular pigment content decreased by 42.06% in the fermenter-optimized bacteria group and by 39.28% in the unoptimized shaking-flask bacteria group (Figure 5). Compared with the algae treated with the unoptimized bacteria, the chlorophyll a of algae treated with the optimized bacteria from the 50 L fermenter decreased by 21.85%. Regarding protein content, a similar decline was observed in both bacterial treatment groups. After 12 h of treatment, the cellular protein content was reduced by 62.43% in the group with fermenter-optimized bacteria and by 1.97% in the group with unoptimized shaking-flask bacteria. When compared to algae treated with unoptimized bacteria, the cellular protein content of algae treated with fermenter-optimized bacteria reduced significantly by 78.3%.

### 3.4. Oxidative Stress and Antioxidant Responses of K. mikimotoi to Optimized Pseudoalteromonas sp. FDHY-MZ2 Culture

**Figure 6:**
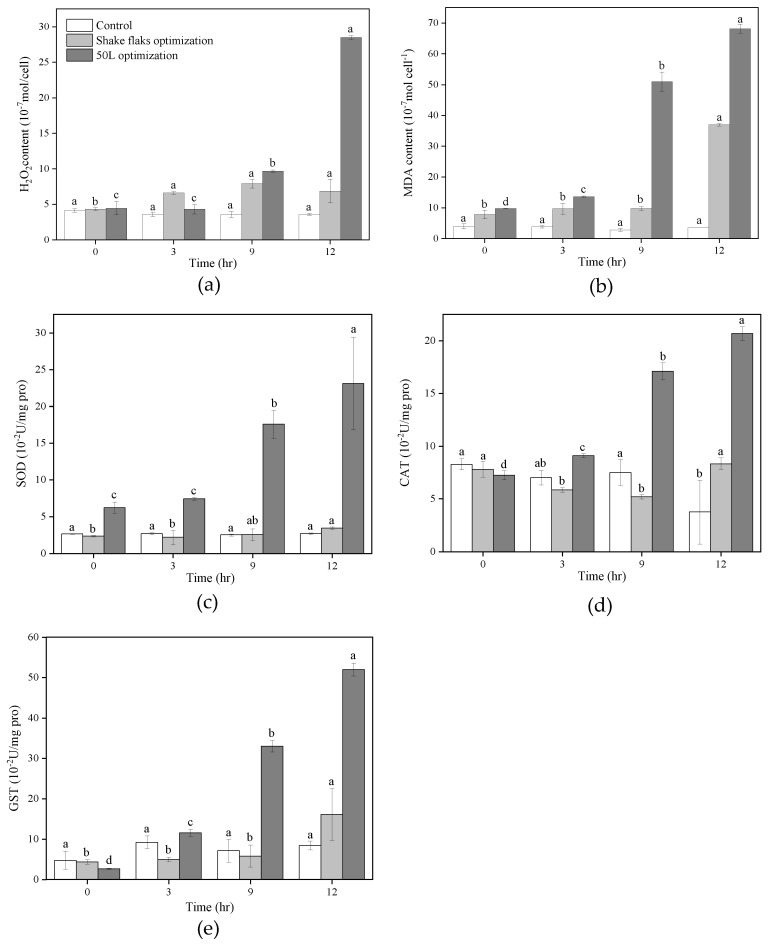
Oxidation and antioxidation traits of *K. mikimotoi* with co-culture of algal cell and different FDHY-MZ2 fermentation broth (shake flask optimization and 50 L optimization). The tested indicator included (**a**) H_2_O_2_; (**b**) MDA; (**c**) SOD; (**d**) CAT; and (**e**) GST. Data represent the mean *± SD (standard deviation)* of triplicate. Different lowercase letters indicate statistically significant differences; the significance level is *p* < 0.05.

The effects of exposure to optimized *Pseudoalteromonas* sp. FDHY-MZ2 on *K. mikimotoi* oxidative stress and antioxidant defense were evaluated. Algal cultures were divided into three groups: those treated with 50 L fermenter optimized bacteria, those treated with unoptimized shaking-flask bacteria, and those without any added bacteria (control) (Figure 6). Oxidative stress in algal cells was assessed by tracking cellular H_2_O_2_ and MDA levels (Figure 6a,b). In the control groups, both H_2_O_2_ and MDA concentrations remained stable throughout the 12 h study.

Both algicidal bacterial treatment groups showed an increase in H_2_O_2_ within the first 3 h of exposure, subsequently maintaining a higher level. Compared to the shaking-flask bacteria treatment group, the fermenter-optimized group showed higher H_2_O_2_ levels. After 12 h of treatment, the H_2_O_2_ concentration in the fermenter-optimized group was 4.14 times that of the shaking-flask bacteria treatment group and 7.97 times that of the control group (Figure 6a). The MDA content in the shaking-flask bacteria treatment group increased during the initial 3 h and remained at a higher level thereafter. In the 50 L fermenter optimized group, MDA content was 3.57 and 1.41 times higher than that of the control group and shaking-flask bacteria treatment group. The content remained at that level until the 9 h time point, before sharply increasing after 12 h of treatment. Finally, the MDA concentration in the fermenter-optimized group was 1.84 times that of the shaking-flask bacteria treatment group and 18.86 times that of the control group (Figure 6b).

The antioxidant defense mechanisms in algal cells were assessed by determining the activity of the enzymes SOD, CAT, and GST [29]. The levels of SOD in both the treatment and control groups remained approximately the same throughout the varying sampling points; however, 12 h post-exposure, there was a slight elevation within the fermenter-optimized treatment group relative to both the shaking-flask bacteria treatment group and the control group (6.75- and 8.63-fold increases, respectively). Within the fermenter-optimized group, CAT activity was initially the same as that in the control group, declining to reach its lowest value at 9 h, followed by a rebound to match the activity level of the shaking-flask bacteria treatment group at the 12 h sampling point. Additionally, the GST assay revealed an increase in enzyme activity observed in both the shaking-flask bacteria treatment group and the fermenter-optimized group throughout the 12 h treatment period, which contrasted with the stable levels in the control group. At the 12 h mark, the GST activity within the 50 L fermenter optimized group was 3.23-fold and 6.17-fold higher than that of the shaking-flask bacteria treatment group and the control group, respectively (Figure 6e).

### 3.5. Optimized Pseudoalteromonas sp. FDHY-MZ2-Induced Photochemical Responses in K. mikimotoi

**Figure 7:**
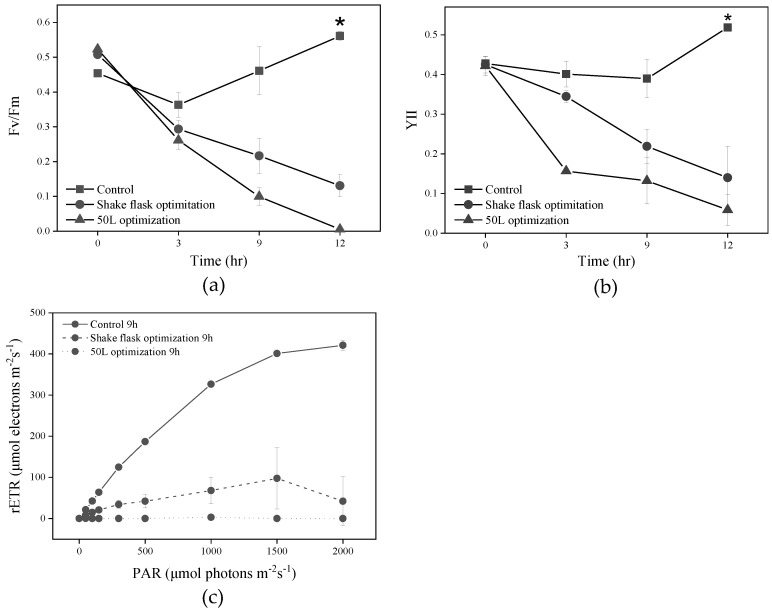
Photochemical traits of (**a**) Fv/Fm; (**b**) YII; and (**c**) rETR in *K. mikimotoi* with co-culture of algal cells and different FDHY-MZ2 fermentation broth (2216E, shake flask optimization, and 50 L optimization). The rETR results were recorded at 9 h. Data represent the mean *± SD (standard deviation)* of triplicate. * means *p* < 0.05, a significant difference.

The impact of optimized *Pseudoalteromonas* sp. FDHY-MZ2 on the photosynthetic activity of *K. mikimotoi* was investigated by measuring the Fv/Fm, YII, and rETR levels across three different culture groups: a group inoculated with bacteria optimized in a 50 L fermenter, a group inoculated with unoptimized bacteria cultivated in a shaking flask, and a control group with no bacteria (Figure 7). A comparative analysis revealed a significant decrease in all three parameters in the treated groups as compared to the control group, a pattern which was particularly pronounced as the duration of treatment increased. Notably, Fv/Fm, rETR, and YII exhibited an even greater decrease with the fermenter-optimized bacteria, indicating a distinct influence of this treatment on the photosynthetic activity of *K. mikimotoi*.

## 4. Discussion

Recently, the global incidence and magnitude of HABs have escalated, prompting researchers to increasingly consider bioagents for HAB control [44,45,46]. Despite the growing discovery of algicidal bacterial strains, scant attention has been devoted to process optimization for their large-scale production. The growth dynamics of bacteria largely hinge on the culture medium and conditions [47]. In this study, the growth of strain FDHY-MZ2, a bacterium shown to have notable algicidal activity against *K. mikimotoi*, was optimized across various scales (a shaking flask, and 5 L and 50 L fermenters). Optimization of bacterial culture conditions subsequently heightened the algicidal activity displayed by *Pseudoalteromonas* SP48 against the algae species *Alexandrium tamarense* within a 5 L fermentation cavity [17]. Our previous work suggested that the strain FDHY-MZ2 has a modest algicidal rate against *Alexandrium* species [36]. The optimization of effective culture conditions for algicidal bacteria has also been reported in other types of strains; for instance, enhanced algicidal activity of *Vibrio brasiliensis* H115 against *Akashiwo sanguinea* [48]. Drawing upon the experimental procedures from these studies, a method of fermentation cultivation specifically tailored towards mitigating *K. mikimotoi* algal blooms was designed, employing strain FDHY-MZ2.

Previous research has demonstrated that when *Pseudoalternators* sp. is cultivated on an unsuitable nutrient-addition medium, it yields cells deficient in algicidal activity, resulting in minimal production of algicidal active chemicals [49]. Consequently, medium optimization has been identified as a crucial strategy for enhancing product yield and making process improvements. During process development, a systematic exploration of medium compositions through various carbon and nitrogen source screenings and combinations can significantly enhance microbial growth, viability, and overall product yield [50]. Nutrient optimization was performed for the algicidal bacterium *Enterobacter* sp. NP23 to improve its activity against several algal species [48]. In this study, shake flask optimization demonstrated that soluble starch was the optimum carbon source for strain FDHY-MZ2, a finding that aligns with a previous report regarding the fermentation of algicidal *Actinomycete* sp. [51]. An orthogonal design was previously employed to optimize the medium composition of *Pseudoalteromonas* SP48 [17]. There is consistency between the optimal nitrogen source identified in their trials and this study (peptone). Orthogonal experiments have been shown to significantly increase bacterial biomass and algicidal efficiency.

During fermentation, microorganisms process substrates into metabolites, leading to changes in the acid–base balance, which can induce stress [52]. The maintenance of pH homeostasis is critical for the functionality and stability of all cellular enzymes [52,53]. In the initial shake flask culture, the optimal pH was determined to be 7.5, reflecting the bacterium’s marine habitat where the estimated seawater pH is approximately 8.0. Extremities on either end of the pH spectrum are known to impede cellular growth, with inhibitory effects apparent under both high and low pH [54]. The constant pH optimization experiment conducted in a 5 L fermenter indicated that the bacteria thrived optimally without modifications to pH, potentially due to the feedback mechanism initiated with the substances secreted by the bacteria, promoting growth. This finding aligns with a similar study that demonstrated that the synthesis of nattokinase by *Bacillus licheniformis* was facilitated with a fermentation process that did not entail constant pH regulation [55].

With the scale-up of fermentation equipment, parameters such as rotational speed, ventilation volume, and feeding methodology require adjustment and improvement [13]. The dissolved oxygen content influences the productivity of bioactive compounds during fermentation [56,57,58]. This parameter is usually correlated with a high-density microbial culture within fermentation systems. Generally, raising the rotational speed increases the dissolved oxygen content, which can influence the synthesis rate of metabolites [59]. The rotational speed is typically adjusted based on both the dissolved oxygen requirements and the biomass [59]. Here, it was determined that while high rotational speeds may initiate an earlier decline, the increased fermenter volume results in a higher dissolved oxygen content. In the 50 L fermenter, the rotational speed was elevated to 450 rpm from 250 rpm in the shake flask; this not only enhanced the biomass but also extended the stable period of fermentation. Consistent with this approach, it has been demonstrated that when scaling up from a bottle to a 50 L fermenter culture, the mixing speed could be adjusted from the initial 200 rpm to 500 rpm [13].

An effective fermentation strategy reduces production costs and enhances productivity [60]. Prior studies have highlighted the importance of the fed-batch strategy for fermentation optimization [60]. For 50 L optimization, supplementing depleted nutrients can mitigate the inhibitory effect of substrates, extend the production period of secondary metabolites, and dilute toxic metabolites [61]. Here, substrate inhibition was seen when only nitrogen sources were supplemented; adding both nitrogen and carbon sources into the culture medium prevented nutritional imbalances. The addition of a complete culture medium resulted in the highest biomass from 68 to 78 h, which remained consistently high (Figure S6); although the addition of a carbon source after 78 h yielded more biomass than the addition of the complete culture medium, it did not result in a significant increase over the biomass obtained from adding the complete medium between 68 and 78 h. To ensure high biomass while reducing operation-associated consumption, the addition of a complete medium was chosen as the optimal condition. A similar fed-batch strategy was reported for the marine fungus *Schizochytrium* sp., which was used to maximize DHA yield [62].

In the 50 L fermenter, the optimal feeding strategy changed to a constant rate feeding of a single carbon source. This difference from the 5 L method might be attributed to the prolonged feeding time under constant feeding, which increased biomass. The bacteria were able to sustain growth through only the increased carbon source, leading to results similar to the study by Ma et al. [19]. The feeding flow rate corresponded closely with the change in dissolved oxygen. Research by Zhang et al. [63] demonstrated that the feeding method and rate can not only alter the carbon source supplement but also affect the level of dissolved oxygen. Through the 5 L optimization experiments, the biomass of the algicidal bacteria increased by 10.92 times, and the algicidal effect was greatly improved. After optimizing the conditions of the 50 L fermentation tank, the algae dissolution rate was increased by 5 times compared with the 5 L optimized fermentation, and the OD_600_ and dry weight increased by 1.65 times and 1.3 times, respectively.

The Fv/Fm and maximum rETR are used to assess photosynthetic efficiency and capacity [64]. These parameters are often disrupted by environmental stresses as well as algicidal bacteria [65,66]. With varying fermentation products, the values of Fv/Fm, YII, and rETR all decreased in algal cells compared with the control group. It is noteworthy that these values decreased even more for *K. mikimotoi* cells treated with the optimized algicidal bacteria from the 50 L fermenter, suggesting that the algal cells’ photosynthetic systems were significantly disrupted by the optimized FDHY-MZ2 fermentation product.

MDA content, an indicator of lipid peroxidation in algal cells, consistently increased during the experiment, suggesting an escalating rate of lipid peroxidation. This implies that the antioxidant system of the algal cells might not be able to clear the excessive ROS efficiently, leading to long-term oxidative damage. This finding aligns with other studies noting increased MDA content in algal cells following exposure to algicidal bacteria [67,68]. In this study, the levels of MDA and H_2_O_2_ decreased with various algicidal bacteria fermentation broths, indicating peroxidation and damage to the algal cell membrane lipids, which produced an abundance of reactive oxygen species. As the fermentation scale increased, the FDHY-MZ2 fermentation broth exhibited a stronger destructive impact on the algal cells; in the 50 L fermenter group, more ROS and MDA were detected, suggesting the algal cells were more seriously damaged. The antioxidant enzymes, SOD and CAT, displayed minimal changes with the addition of fermentation broth; the levels of these two enzymes in the shaking flask and 50 L optimized groups were the same at most sampling time points. This contrasts with the significant accumulation of oxidative substances in the 50 L optimized treatment group, where a large amount of accumulated peroxides could not be removed, potentially resulting in oxidative damage to the algal cells [68]. This may be the main reason for the substantial improvement seen in the algal lysis performance of the 50 L optimized fermentation broth.

Previous cultivation of algicidal bacteria has primarily been confined to small-scale fermentation optimization (5 L fermenters), with few attempts made at pilot fermentation. In this study, the process was successfully scaled and both the biomass and algicidal activity were increased, laying solid groundwork for potential large-scale fermentation. In the context of HAB management utilizing algicidal agents, it is crucial to produce a substantial biomass of algicidal bacteria and maintain high algal dissolution capacity per unit volume. Increased biomass allows for the potential immobilization of the bacteria, enabling continuous release of algicidal compounds over extended periods [5]. This strategy has been applied for multiple bacteria species employed for harmful algae removal [69,70] and drives preventive mechanisms against HABs within specified areas. In addition, high algicidal activity per unit volume provides these bacteria with a strategic advantage in the creation of effective algicidal agents capable of neutralizing algae in areas of dense HAB manifestation [22]. The method used in this study can also serve as a reference for optimizing the fermentation process of various algicidal bacteria, allowing research to get closer to the goal of HAB eradication. By probing the effects of algicidal bacteria on the physiology and biochemistry of *K. mikimotoi*, this study provides a critical theoretical basis for the algicidal actions of the bacteria, which could assist in further isolation and characterization of specific algicidal compounds that can degrade algal toxins or inhibit their release into the environment.

## 5. Conclusions

The algicidal bacterium *Pseudoalteromonas* sp. FDHY-MZ2 effectively inhibited the growth of the HAB species *K. mikimotoi*. Through the optimization of a shake flask culture to a 5 L and a 50 L fermenter, the optimal culture medium and cultivation conditions for the 50 L fermentation of this strain were obtained. The optimal culture medium was determined to be a 2216E basal medium with 1.5% soluble starch and 0.5% peptone. The optimal conditions were 66 h of fermentation at 20 °C and 450 rpm, with a 30 L/min ventilation volume, 3% (*v*/*v*) seeding proportion, and constant flow of high-concentration soluble starch at 30 h. The biomass of the algicidal bacteria obtained by using this strategy increased by 10.92 times, and the dosage of algicidal bacteria that could kill 100% of *K. mikimotoi* within 24 h decreased from 1% to 0.025%. Under the influence of this algicidal agent, there were considerable decreases in the chlorophyll and protein levels in the algal cells. Damage was more pronounced to the photosynthetic system, leading to an increase in the production of peroxidative substances, while the enzyme activities related to peroxide clearance remained relatively static. This study provides both a demonstration and theoretical basis for the application of algicidal bacteria in HAB management.

## Figures and Tables

**Table 1:** L_9_(4^3^) Orthogonal design and results of FDHY-MZ2 shake flask optimization.

Experiment No.	Carbon Sources (A,%)	Nitrogen Sources (B,%)	pH (C)	Time (D,h)	OD_600_
1	1 (0.5)	1 (0.5)	1 (7.5)	1 (24)	3.88
2	1 (0.5)	2 (1.0)	2 (8.0)	2 (36)	5.64
3	1 (0.5)	3 (1.5)	3 (8.5)	3 (48)	6.15
4	2 (1.0)	1 (0.5)	2 (8.0)	3 (48)	7.38
5	2 (1.0)	2 (1.0)	3 (8.5)	1 (24)	3.75
6	2 (1.0)	3 (1.5)	1 (7.5)	2 (36)	5.78
7	3 (1.5)	1 (0.5)	3 (8.5)	2 (36)	6.10
8	3 (1.5)	2 (1.0)	1 (7.5)	3 (48)	7.71
9	3 (1.5)	3 (1.5)	2 (8.0)	1 (24)	3.54
K1	15.68	17.36	17.36	11.17	
K2	16.90	17.10	16.56	17.52	
K3	17.35	15.46	16.00	21.23	
k_1_	5.23	5.79	5.79	3.72	
k_2_	5.63	5.70	5.52	5.84	
k_3_	5.78	5.15	5.33	7.08	
R	0.56	0.63	0.46	3.35	
Factor order	D > B > A > C				
Optimal level	A_3_	B_1_	C_1_	D_3_	
Optimum combination	D_3_B_1_A_3_C_1_				

K represents the sum of three OD_600_ values at the same level of a certain factor, and k is the corresponding K/3. R represents the level difference between k1, k2, and k3 under the same factor. Higher values of R signify a pronounced impact on the test results, with the optimal level correlating to the factor yielding the highest value of k.

## Data Availability

All data were included in the paper.

## Supplementary Materials

The following supporting information can be downloaded at: https://www.mdpi.com/article/10.3390/biology12111447/s1, Table S1: L_9_(4^3^) Orthogonal design of FDHY-MZ2 shake flask optimization; Table S2: Optimized conditions for different fermenter sizes and results after 66 h of fermentation; Figure S1: Influence of different culture conditions on strain FDHY-MZ2 growth and algicidal effect; Figure S2: Influence of different medium components on strain FDHY-MZ2 growth and algicidal effect; Figure S3: Results of OD_600_ and dry weight of strain FDHY-MZ2 with 2216E medium and optimized cultivate (medium and conditions); Figure S4: Changes of fermentation indexes: OD_600_ and algicidal rate of FDHY-MZ2 on *Karenia mikimotoi* at different rotating speeds (150 rpm, 200 rpm, 300 rpm) in 5 L fermenter; Figure S5: Changes of fermentation indexes: OD_600_ and algicidal rate of FDHY-MZ2 on *Karenia mikimotoi* at different constant pH (pH6.5, pH7.5, pH8.5) in 5 L fermenter; Figure S6: Changes of fermentation indexes: OD_600_ and algicidal rate of FDHY-MZ2 on *Karenia mikimotoi* with different feeding strategies in 5 L fermenter; Figure S7: Algae dissolution experiment with different volume ratios of bacteria and algae after optimization of feeding strategy; Figure S8: 50 L fermenter condition optimization; Figure S9: Changes in dissolved oxygen (DO) at different rotation speed in 50 L fermenter.
