# Supplementary material for: Pilot-Scale Fermentation of Pseudoalteromonas sp. Strain FDHY-MZ2: An Effective Strategy for Increasing Algicidal Activity
Source: Biology (Basel). 2023 Nov 17;12(11):1447. doi: 10.3390/biology12111447 (PMC10669318; doi:10.3390/biology12111447)
Supplement: Supplementary file 1 [file biology-12-01447-s001.zip › biology-2623915-supplementary.pdf]

Table S1. L_9 (4^3) Orthogonal design of FDHY-MZ2 shake flask optimization

Experiment no.	Carbon sources(A,%)	Nitrogen sources(B,%)	pH(C)	Time(D,h)
1	1(0.5)	1(0.5)	1(7.5)	1(24)
2	1(0.5)	2(1.0)	2(8.0)	2(36)
3	1(0.5)	3(1.5)	3(8.5)	3(48)
4	2(1.0)	1(0.5)	2(8.0)	3(48)
5	2(1.0)	2(1.0)	3(8.5)	1(24)
6	2(1.0)	3(1.5)	1(7.5)	2(36)
7	3(1.5)	1(0.5)	3(8.5)	2(36)
8	3(1.5)	2(1.0)	1(7.5)	3(48)
9	3(1.5)	3(1.5)	2(8.0)	1(24)

Table S2. Optimized conditions for different fermenter sizes and results after 66 hours of fermentation

Fermenter size	Optimition conditions					Results(afer 66 h fermentation)		
	Rotational speeds	Ventilation volume	Inoculums level	Feeding strategy	pH	OD_{600}	Dry weight	Bacteria-algae ratio(achieve 100% algicidal rate in 24 h)
**5 L**	300 rpm	2 L/min	5 %	Feeding full culture medium	Initial pH 7.5 and no subsequent control	10.21	12.28 g/L	0.05%(v/v)
**50 L**	450 rpm	30 L/min	3 %	Constant feeding soluble starch	Initial pH 7.5 and no subsequent control	35.05	27.70 g/L	0.025%(v/v)

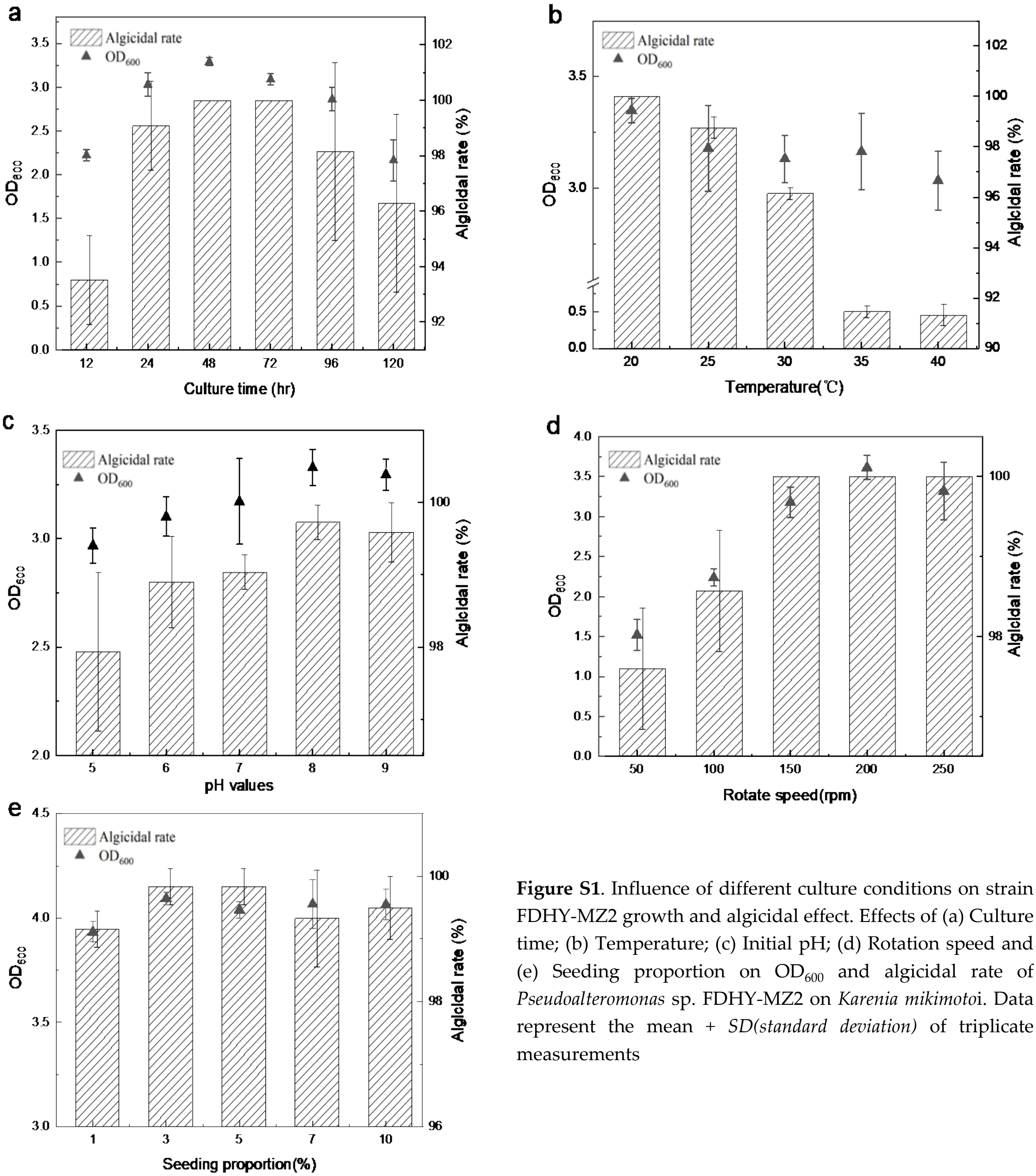

Figure S1. Influence of different culture conditions on strain FDHY-MZ2 growth and algicidal effect. Effects of (a) Culture time; (b) Temperature; (c) Initial pH; (d) Rotation speed and (e) Seeding proportion on OD_{600} and algicidal rate of *Pseudoalteromonas* sp. FDHY-MZ2 on *Karenia mikimoto*i. Data represent the mean + *SD(standard deviation)* of triplicate measurements

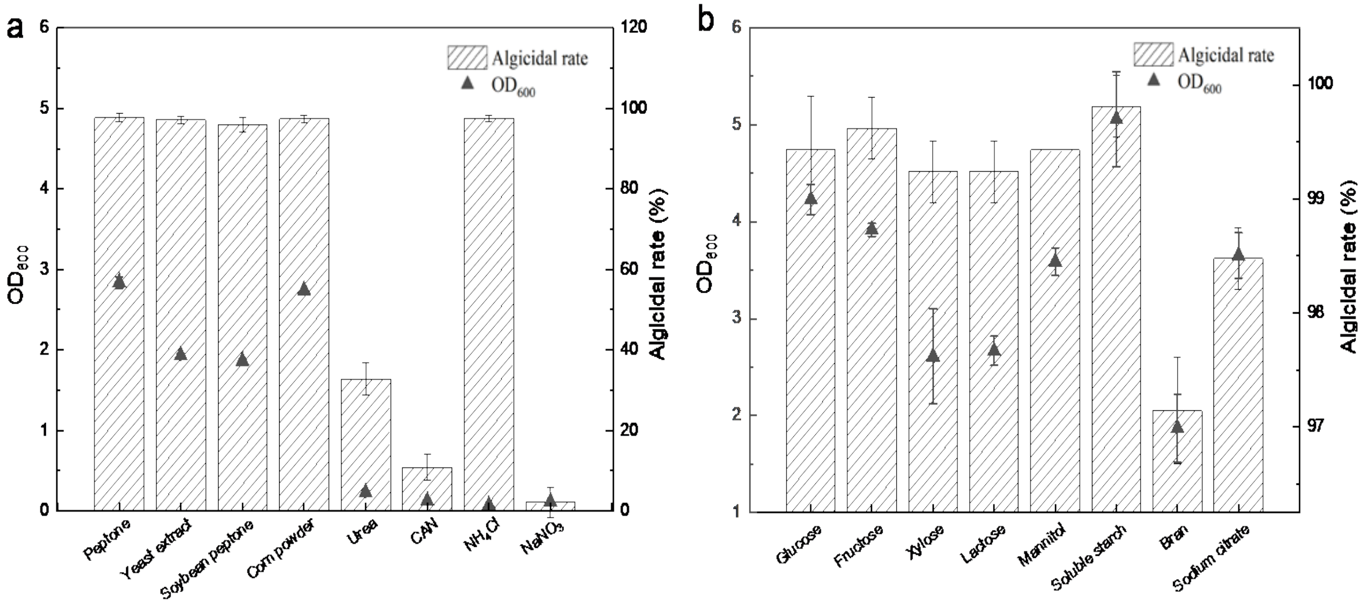

Figure S2. Influence of different medium components on strain FDHY-MZ2 growth and algicidal effect. Effects of (a)Carbon sources, (b)Nitrogen sources on OD_{600} and algicidal rate of *Pseudoalteromonas* sp. FDHY-MZ2. Data represent the mean + *SD(standard deviation)* of triplicate measurements

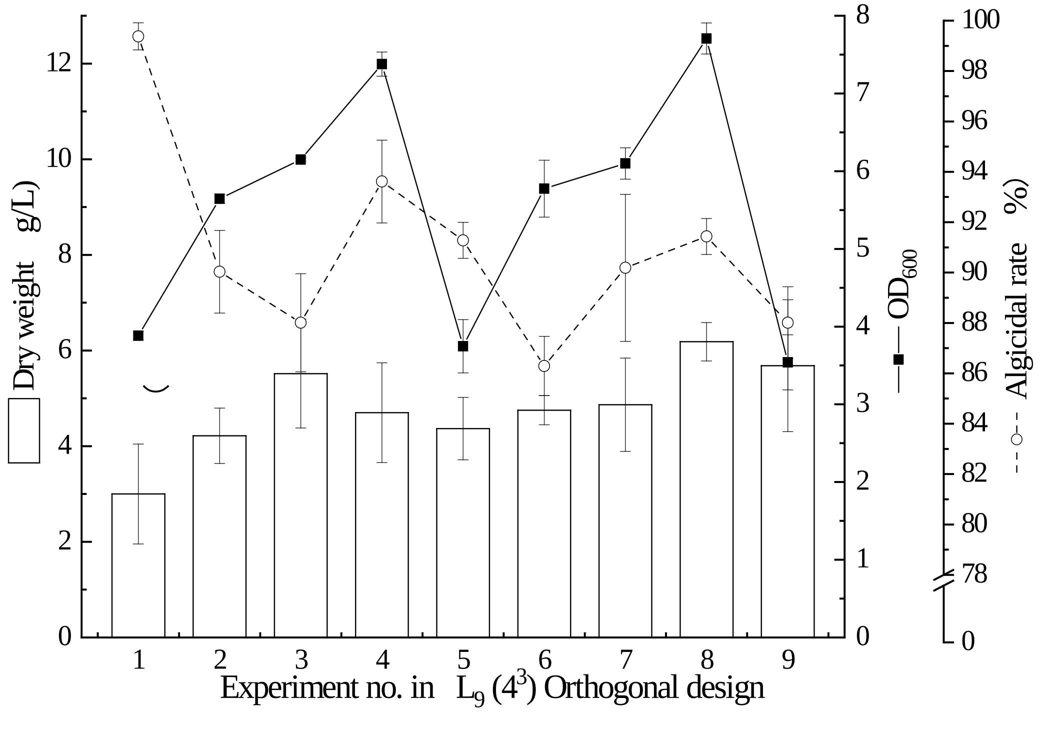

Figure S3. Results of OD_{600} and dry weight of strain FDHY-MZ2 with 2216E medium and optimized cultivate (medium and conditions). Data represent the mean + *SD(standard deviation)* of triplicate

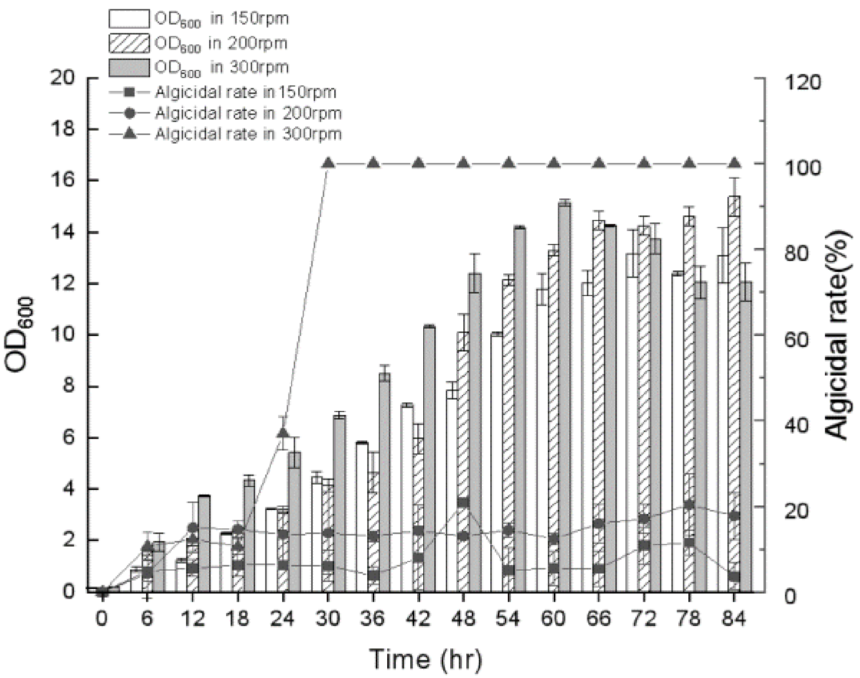

Figure S4. Changes of fermentation indexes: OD_{600} and algicidal rate of FDHY-MZ2 on *Karenia mikimotoi* at different rotating speeds(150 rpm,200 rpm,300 rpm) in 5 L fermenter. Data represent the mean + *SD(standard deviation)* of triplicate measurements (n=3)

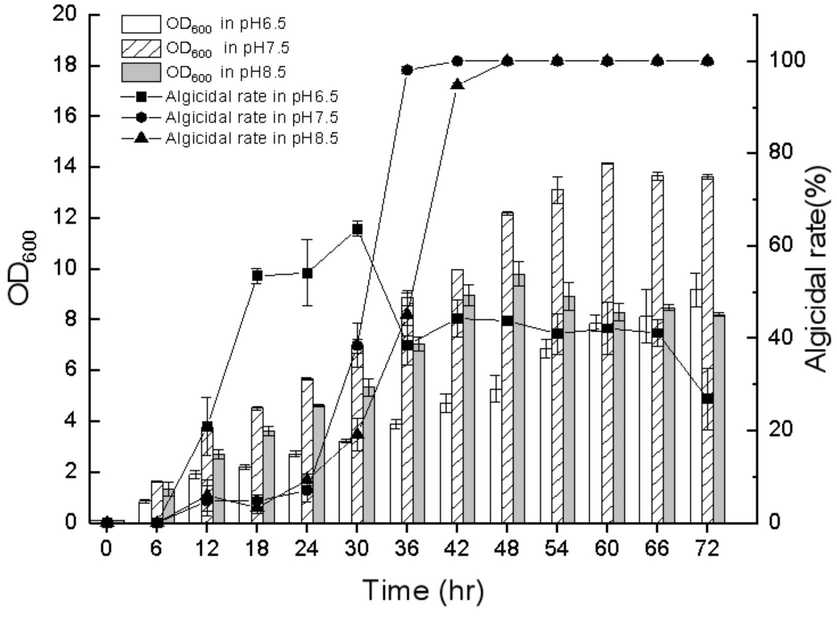

Figure S5. Changes of fermentation indexes: OD_{600} and algicidal rate of FDHY-MZ2 on *Karenia mikimotoi* at different constant pH(pH6.5,pH7.5,pH8.5) in 5 L fermenter. Data represent the mean + *SD(standard deviation)* of triplicate measurements (n=3)

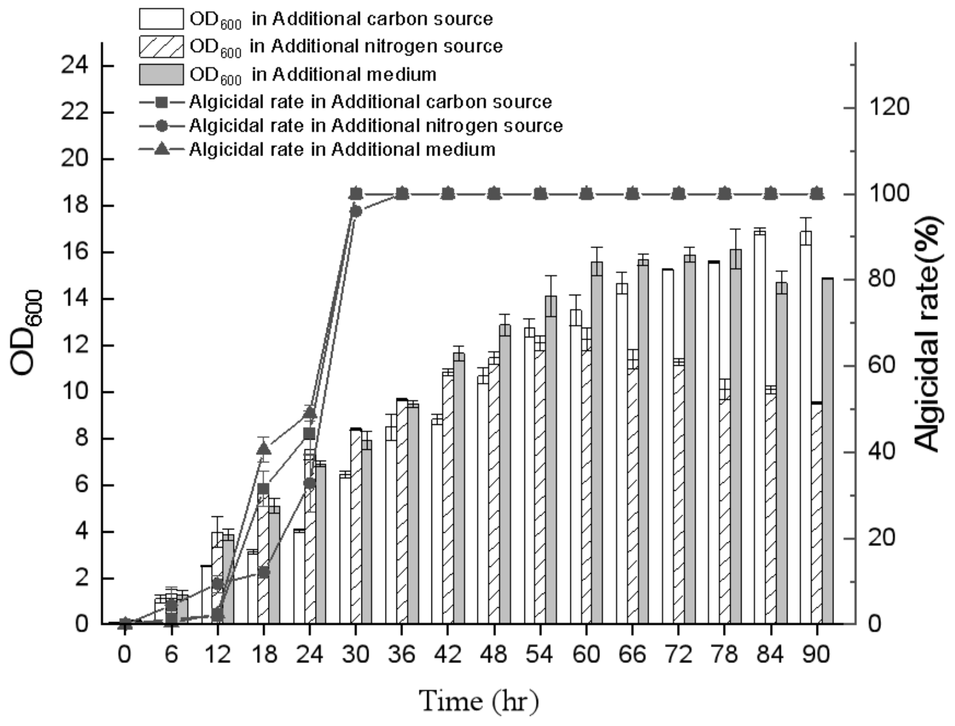

Figure S6. Changes of fermentation indexes: OD_{600} and algicidal rate of FDHY-MZ2 on *Karenia mikimotoi* with different feeding strategies in 5 L fermenter. Data represent the mean + *SD(standard deviation)* of triplicate measurements

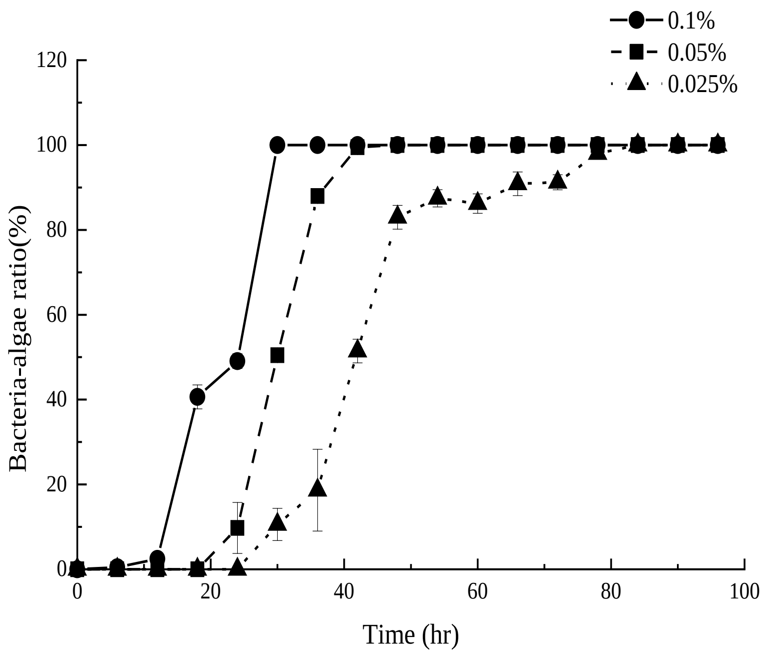

Figure S7. Algae dissolution experiment with different volume ratios of bacteria and algae after optimization of feeding strategy. Data represent the mean + *SD(standard deviation)* of triplicate measurements

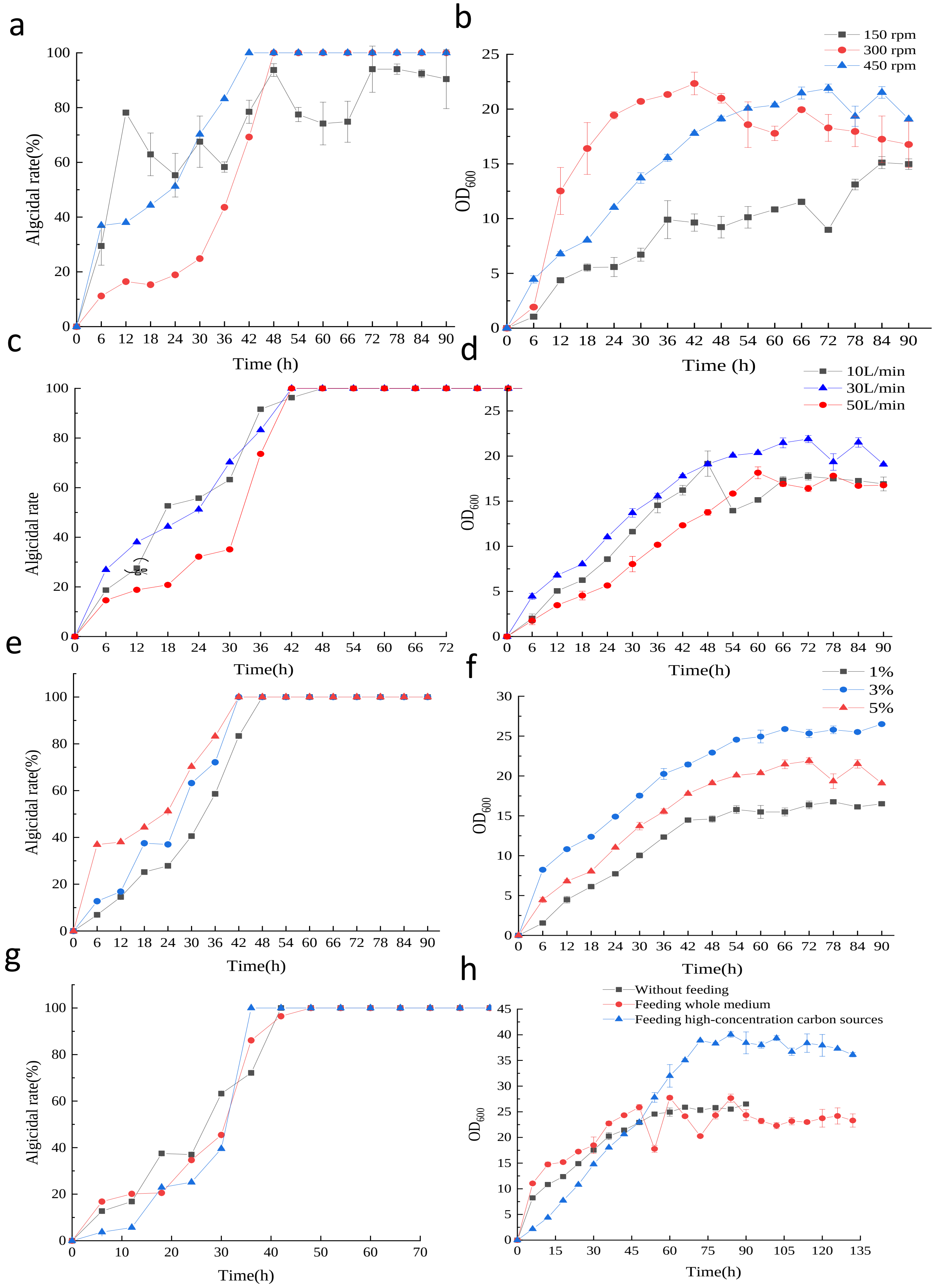

Figure S8. 50 L fermenter condition optimization. a-b, rotational speeds optimization using (a) algicidal rate and (b) OD_{600} value as indicators. c-d, ventilation volume optimization using (c) algicidal rate and (d) OD_{600} value as indicators. e-f, inoculums level optimization using (e) algicidal rate and (f) OD_{600} value as indicators. g-h, feeding strategy optimization using (g) algicidal rate and (h) OD_{600} value as indicators. Error bars depict standard deviations from triplicate culture.

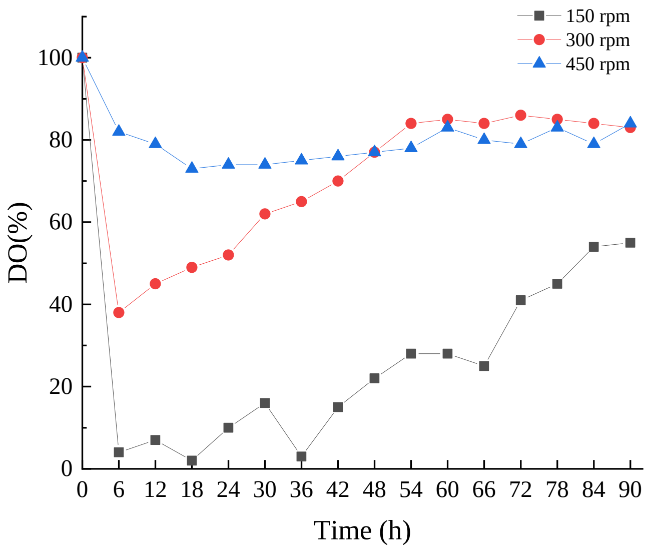

Figure S9. Changes in dissolved oxygen(DO) at different rotation speed in 50 L fermenter.
